# Precise engineering of hybrid molecules-loaded macromolecular nanoparticles shows *in vitro* and *in vivo* antitumor efficacy toward the treatment of nasopharyngeal cancer cells

**DOI:** 10.1080/10717544.2021.1902022

**Published:** 2021-04-19

**Authors:** Dongmei Liu, Wenguang Zhang, Xinju Liu, Rongliang Qiu

**Affiliations:** a Department of Radiation Oncology, Henan Cancer Hospital, Affiliated Cancer Hospital of Zhengzhou University, Zhengzhou, China; b Department of Interventional Radiology, The First Affiliated Hospital of Zhengzhou University, Zhengzhou, China

**Keywords:** Combinational delivery, nasopharyngeal cancer, apoptosis, *in vivo* antitumor efficacy

## Abstract

Cancers continue to be the second leading cause of death worldwide. Despite the development and improvement of surgery, chemotherapy, and radiotherapy in cancer management, effective tumor ablation strategies are still in need due to high cancer patient mortality. Hence, we have established a new approach to achieve treatment-actuated modifications in a tumor microenvironment by using synergistic activity between two potential anticancer drugs. Dual drug delivery of gemcitabine (GEM) and cisplatin (PT) exhibits a great anticancer potential, as GEM enhances the effect of PT treatment of human cells by providing stability of the microenvironment. However, encapsulation of GEM and PT fanatical by methoxypoly(ethylene glycol)-block-poly(D, L-lactic acid) (PEG-PLA in termed as NPs) is incompetent owing to unsuitability between the binary Free GEM and PT core and the macromolecular system. Now, we display that PT can be prepared by hydrophobic coating of the dual drug centers with dioleoylphosphatidic acid (DOPA). The DOPA-covered PT can be co-encapsulated in PLGA NPs alongside GEM to stimulate excellent anticancer property. The occurrence of the PT suggestively enhanced the encapsulations of GEM into PLGA NPs (GEM-PT NPs). Further, the morphology of GEM NPs, PT NPs, and GEM-PT NPs and nanoparticle size was examined by transmission microscopy (TEM), respectively. Furthermore GEM-PT NPs induced significant apoptosis in human nasopharyngeal carcinoma CNE2 and SUNE1 cancer cells by *in vitro*. The morphological observation and apoptosis were confirmed by the various biochemical assays (AO-EB, nuclear staining, and annexin V-FITC). In a xenograft model of nasopharyngeal cancer, this nanotherapy shows a durable inhibition of tumor progression upon the administration of a tolerable dose. Our results suggest that a macromolecular hydrophobic and highly toxic drug can be rationally converted into a pharmacologically efficient and self-deliverable of nanotherapy.

## 1. Introduction

Combination therapy can be performed via coadministration of a supplementary cancer drug along with a sensitizer. The interfaces within potential anticancer drugs rely on the dose ratios between the two medications and can be potentially incompatible. Consequently, the importance of preserving a beneficial ratio to maintain a synergistic relationship between two drugs through nanoparticles (NPs) formulations cannot be ignored (Gadde, 2015; Li & Finley, 2018; Xiao et al., 2018; Wu et al., 2020). The procedure of encapsulating several anticancer drugs in individual NPs has proved to be problematic because the drugs have to preserve their important physicochemical properties (Huang et al., 2016; Emiliano & Almeida-Amaral, 2018; Hong et al., 2020; Sharifi et al., 2020). Hence, nanoformulations that are prepared by encapsulating numerous medications with varied physicochemical belongings while preserving controlled ratios are preferred for drug delivery within the body tissues (Sulthana et al., 2017; Zhang & Tung, 2017; Wang et al., 2018; Laroui et al., 2019; Nejabat et al., 2020).

NP-based drug delivery systems have been developed as a valuable system among other important methods for improved malignancy treatment (Ruan et al., 2019; Yang et al., 2019; Yu et al., 2020). Appropriately, structured NPs can isolate the medications from the circulatory system and evade being eliminated by the renal system (Zhou et al., 2014; Chung et al., 2017; Zhou et al., 2020). These NPs have an advanced system to deliver anticancer medications to targeted locations and decrease nonspecific harm to the target tissues, brought about through enhanced permeability and retention (EPR) effects. Moreover, NP frameworks offer stable watery scattering of medications by surface adjustment and shield medications from degradation, resulting in improved anticancer action (Hu et al., 2012; Johnson et al., 2020; Singhai & Ramteke, 2020).

Platinum metal complexes turn out a mainstay in cancer treatments and encompass some of the most powerful and progressive chemotherapeutic drugs. Despite surgical removal of tumors and various therapies scilicet radiation, chemo, immune, hormone, stem cell, precision medicine etc., shield people to stop dying from cancer, highly and inevitably used platinum chemotherapy saves 50–70% of all patients’ life howbeit with few drawbacks and side effects (Wang et al., 2017; Follmann et al., 2018; Ketabat et al., 2019; Ding et al., 2020). Mitigating efforts to vanquish the drawbacks triggered the exploration of more potent metallodrugs concerned with least toxicity by cancer cell selectivity, structural diversity, redox activity with amicable biochemical (biomimicking ability and ligand exchange kinetics) properties (Huxford-Phillips et al., 2013; Wang et al., 2017; Agrawal et al., 2018; Tsakiris et al., 2019). At right time after some of the platinum compounds imprinted their promising *in vitro* anticancer and *in vivo* antitumor properties in the frontline of anticancer metallotherapeutics by addressing the aforementioned criterions with different modes of anticancer activities (Kim & Lim, 2002; Liu et al., 2019; Lin et al., 2021).

As an oral multikinase inhibitor, gemcitabine (GEM) provides antiangiogenic activity in various tumor types by the inhibition of vascular endothelial growth factor receptors (VEGFR), tyrosine kinase with immunoglobulin and epidermal growth factor homology domain 2 (TIE-2), platelet-derived growth factor receptor-β (PDGFR-β), and fibroblast growth factor receptor (FGFR) (Zhang et al., 2013; Konstantinopoulos et al., 2020; Thompson et al., 2020; Yalcin et al., 2020). The activity is correlated with suppression of cell proliferation, and induction of apoptosis by the inhibition of oncogenic kinases (KIT, RET, RAF-1, BRAF, and mutant BRAF). The US Food and Drug Administration (FDA) approved it for treating gastrointestinal stromal or metastatic colorectal cancer (mCRC). In 2017, FDA approved GEM for a second-line therapy in previously sorafenib treated HCC patients (Sandblom et al., 2019; Unnam et al., 2019; Wu et al., 2020). Some commercial products of non-biodegradable microspheres have come into the market already such as DC Beads and Hepasphere™ for TACE. Drugs especially the positively charged were usually shielded by ion exchange approach, and the drug elution kinetics were determined by the ionic environment of physiological fluids (Jiang et al., 2019; Yao et al., 2019; Fazio, 2020).

L-DOPA (L-3,4-dihydroxyphenylalanine) is a catecholaminedrug that plays important roles in biochemistry and medicinalchemistry. It can be converted to dopamine by dopa-decarboxylaseto increase dopamine level in brain and can also cross the blood–brain barrier, whereas dopamine itself cannot. This has model-DOPA the most effective drug for the treatment of various disease. Abnormal concentrations of L-DOPA in biological fluids (e.g. urine, plasma and serum) can be used for the diagnosis of Parkinson’s disease and for evaluating the activity of the sympathetic nervous system.

In this work, we have described a nanoplatform formed by encapsulation of two potential drugs into PLGA nanoparticles (GEM-PT NPs) via a nanoprecipitation method. Furthermore, *in vitro* cytotoxicity of the drug-loaded NPs was examined in human Nasopharyngeal (CNE2 and SUNE1 cancer cells using an MTT assay. Additionally, we examined morphological changes in the treated cells by dual staining (AO-EB) and nuclear staining methods. Apoptosis was confirmed by the flowcytometry analysis. To establish the potential of this GEM-PT NPs fabrication to be translated to the clinic, we evaluated the antitumor efficacy in mouse models of human CNE2 tumor xenografts. Our NP-mediated delivery platforms provide a simple, broadly applicable strategy to effectively enhance the potency and safety of molecularly targeted agents that have previously been limited to tumor administration.

## 2. Materials and methods

### 2.1. Materials

PT and GEM were purchased from TCI (Shanghai, China). Hydrolyzed Polyvinyl alcohol (PVA, 85–90%, Mol. Wt of 30 K–50K Da) were obtained from TCI, China. PLGA polymers (monomer ratio 50:50; MW 7 K Da) was acquired from J&K, China.

### 2.2. Methods

#### 2.2.1. Encapsulation of GEM and PT in GEM-PT NPs

An oil/water solvent evaporation technique adapted to encapsulation of PT and GEM in PLGA-NPs (Gupta et al., 2017; Sani et al., 2019; Fu et al., 2020). Briefly, DOPA-coated PT (50 µg) cores and GEM (50 µg) were added to a PLGA-NP solution in CHCl_3_ (100 mg in 350 µl). The emulsified 9% PVA was mixed into chloroformic solution in 3 mL PBS solutions. The emulsions were stirred for 24 h, and evaporate the organic solvents. PT-loaded and GEM-loaded PLGA nanoparticles (GEM-PT NPs) were kept at −20 °C to be used for future studies.

A water/oil/water double emulsion solvent evaporations technique were used to fabricate the PLGA-NPs containing DOPA-coated PT, GEM. Briefly, TMR-dextran (200 µl) was blended into a PT and GEM polymeric solutions in CHCl_3_ with sonication’s. These emulsions were consequently blended in PVA-PBS solutions, left for solvents evaporations. The emulsions were stirred for 24 h, and evaporate the organic solvents.

### 2.3. Examination of *in vitro* drug release

Assessment of *in vitro* drug release kinetics was performed using a dialysis diffusion technique. GEM-PT NPs (3 ml), and PT and GEM (0.1 mg/ml equivalent concentration) solutions were placed into the end-wrapped dialysis covers. Next, they were retained into 20 ml of discharging medium comprising 0.2% Tween-80 in PBS pH 7.4. By stirring at 100 rpm on a detour shakers at 37 °C, the drug release medium was removed and an equivalent size of new medium was added. The drug-releasing profiles of PT and GEM were examined using an UV − vis spectrometer (Huxford-Phillips et al., 2013; Huang et al., 2017; Han et al., 2018).

### 2.4. *In vitro* cytotoxicity

Cancerous CNE2, SUNE1, and non-cancerous HUVEC cells were obtained from the Cell Bank of Beijing. The cells were maintained in RPMI 1640 culture and Dulbecco’s modified Eagle’s (DMEM) medium supplemented with 10% fetal bovine serum (FBS) and 100 ml^−1^ penicillin. Then, Cancerous CNE2, SUNE1, and noncancerous HUVEC cells were incubated in a humid atmosphere with 5% CO_2_ at 37 °C. In vitro biochemical staining was obtained from Cell Signaling (China).

### 2.5. MTT assay

CNE2 and SUNE1 cells were cultured in 96-well plates (4000 cells per well) and incubated for 24 h at 37 °C. Free PT, Free GEM, PT NPs, GEM NPs, and GEM-PT NPs were well dissolved in DMSO and the final contents of DMSO were less than 0.2% (v/v) to avoid the solvent impacting cell viability. Then the cells were treated with various concentrations of Free PT, Free GEM, PT NPs, GEM NPs, and GEM-PT NPs for 24 h. Experiments were performed in triplicate and the medium without the samples were served as the control. After 24 h, 30 μL of 3-[4,5-dimethylthiazol-2-yl]-3,5-diphenyl tetrazolium bromide (MTT) in phosphate buffered saline solution at a concentration of 5 mg mL^−1^ was added into each well and incubated at 37 °C for 5 h. Then the medium with MTT was removed and 100 μL of DMSO was added to dissolve the formazan crystals formed. The absorbance of each sample at 492 nm on a microplate reader (Multiskan FC, Thermo Scientific). Cell viability was calculated as follows: cell viability (%) = [absorbance of each well/absorbance of control well] × 100. Graph was plotted between % of cell inhibition and concentration of the test samples. From this plot, the IC_50_ value was calculated.

### 2.6. Apoptotic staining

The morphological changes of the CNE2 cells were examined by biochemical staining, including acridine orange-ethidium bromide (AO-EB) and Hoechst 33344 staining (Mohamed Subarkhan et al., 2016; Subarkhan & Ramesh, 2016; Mohan et al., 2018; Balaji et al., 2020; Sathiya Kamatchi et al., 2020). After incubating for 24 h, the cells were seeded at a concentration of 1 × 10^4^ onto 48-well plates. The cells were treated with Free PT, Free GEM, PT NPs, GEM NPs, and GEM-PT NPs at 2.5 µM concentration for 24 h. On the following day, the staining solution was added. After incubating the plates with the staining solution, the plates were washed with PBS three times. Images were obtained using a fluorescence microscope (Accu Scope EXI-310) at a magnification of 20×.

### 2.7. Flow cytometry/annexin V-PI staining

The flow cytometry examination was examined by using the Apoptosis Detection Kit of fluoresceinisothiocyanate (FITC) (Cell Signaling, China) utilized to confirm the apoptotic ratio of CNE2 cells. The cells were treated with Free PT, Free GEM, PT NPs, GEM NPs, and GEM -PT NPs at 2.5 µM concentrations for 24 h. The cells were washed thrice by using trypsin, and suspended in 1 × binding buffer (500 μL) with FITC Annexin V (5 μL) and of PI (10 μL). After 20 min incubation, the samples were analyzed by flow cytometry. The obtained results were investigated with the BD FACS CantoTM II flow cytometer.

### 2.8. Evaluation of the *in vivo* drug toxicity

The *in vivo* drug toxicity was investigated in ICR mice (4–5 weeks old). Healthy ICR mice were randomly divided into five groups (*n* = 10 mice per group). Drugs were injected through the tail vein on days 0, 3, and 6. Mice were injected with Free PT (2.5 and 5 mg/kg, Ciaplatin equivalent dose), free GEM (2.5 and 5 mg/kg), PT NPs (2.5 and 5 mg/kg), GEM NPs (2.5 and 5 mg/kg), and PT-GEM NPs (2.5, 5, and 10 mg/kg). Saline were injected as a control. The body weights of the mice were recorded every 3 days.

### 2.9. Histologic analysis

For histological analysis, the organs from the sacrificed mice were excised at the end of the treatments with various drugs. After being fixed in 4% formaldehyde and embedded in paraffin, the tumor tissues and organs were further sectioned into 5 μm slices for hematoxylin and eosin (H&E, Sigma) staining. The H&E-stained tissues were imaged by fluorescence microscopy (Olympus, IX71).

### 2.10. *In vivo* antitumor activity

All animal experiments were approved by the Ethics Committee of the Department of Radiation Oncology, Affiliated Cancer Hospital of Zhengzhou University, Henan Cancer Hospital, Zhengzhou, Henan 450008, China in accordance with the guidelines on animal care and use (File No: 2018-3). BALB/c nude mice (4–5 weeks old) were used for the evaluation of the antitumor activities of the nanotherapies. The human prostate cancer cell line CNE2 was grown to 80% confluence in 90 mm tissue culture dishes. After cell harvesting, the cells were resuspended in PBS at 4 °C to reach a final concentration of 2.5 × 10^7^ cells/mL. The right flanks of the BALB/c nude mice were subcutaneously injected with 200 μL of a cell suspension containing 5 × 10^6^ cells. At 14 days after implantation, the tumors reached approximately 60 mm^3^ in volume, and then the animals were randomly divided into five groups (*n* = 7 mice per group). Mice bearing CNE2 tumor xenografts were injected intravenously with samples solutions (Free PT at 2.5 mg/kg, Free GEM at 2.5 mg/kg, PT NPs at 5 mg/kg, GEM NPs at 5 mg/kg, and PT-GEM NPs at 10 mg/kg) three times on days 0, 3, and 6. Saline were also injected as a control. Tumor volumes and body weights were monitored and recorded for 33 days. The lengths (L) and widths (W) of the tumors were measured with calipers, and the tumor volume was calculated by the following formula: *V* = (*L* × *W*_2_)/2, where W is shorter than L. Mice were sacrificed by CO_2_ inhalation at the endpoint of the study.

### 2.11. Data analysis

The data analysis of different groups was conducted with one-way ANOVA in GraphPad Prism 5 software. The significant level was considered at *p* < .05 and greatly significant at *p* < .001. All data are presented as mean ± SD. (Unless otherwise stated, *n* = 3).

## 3. Results and discussion

### 3.1. Structural morphology and characterization

**Figure 1.**
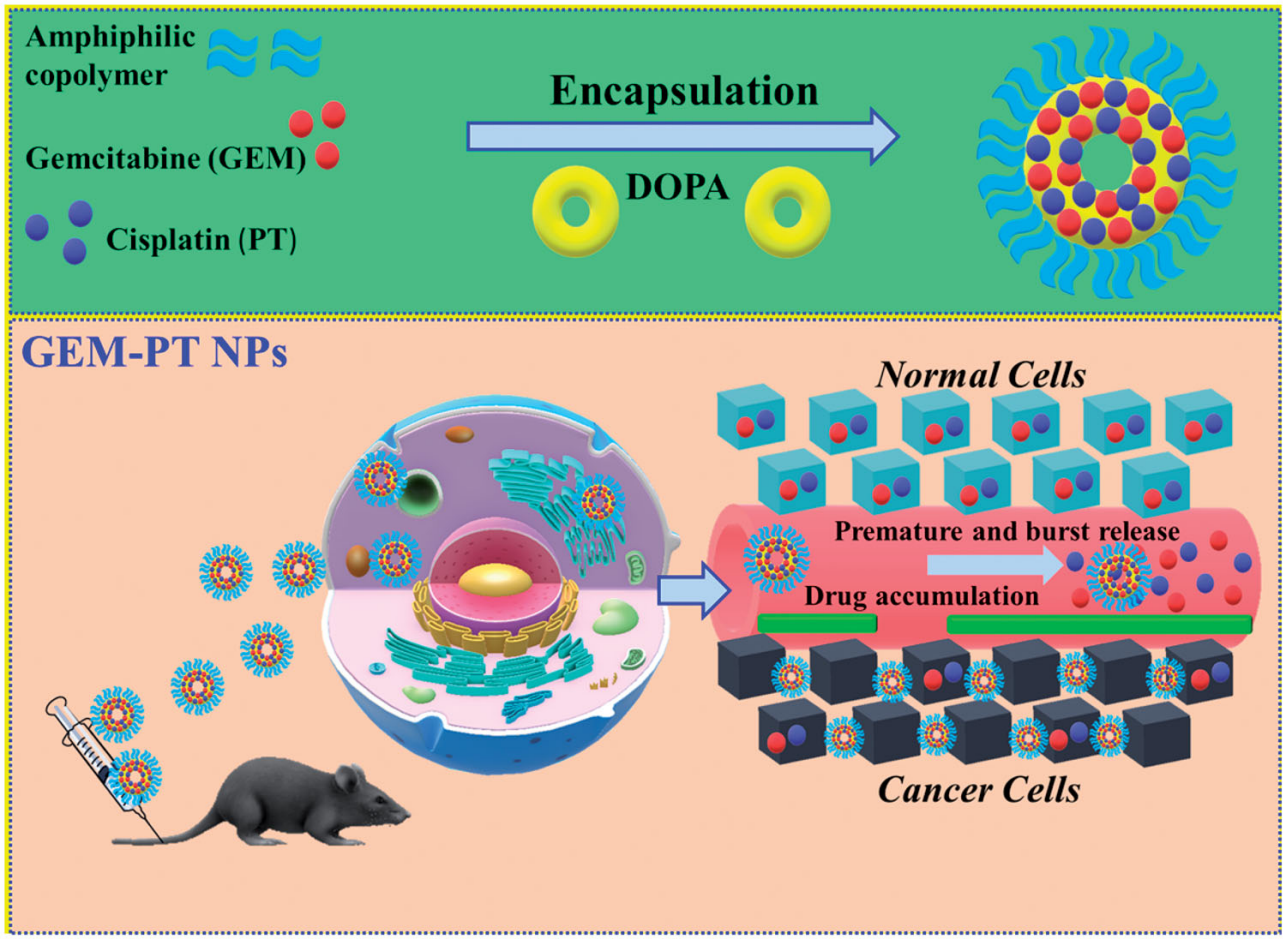
A graphic representation of the encapsulation of GEM and PT into amphiphilic polymers to form GEM-PT NPs for the treatment of cancer therapy.

Our achievement in proficiently stacking of cisplatin (PT) and GEM rafenib(GEM) into PLGA-NPs (designated as GEM-PT NPs) proposals another chance to co-deliver two medications for blend treatment (Shen et al., 2011; Margiotta et al., 2016; Mohamed Subarkhan et al., 2019). For instance, hydrophobic PT and GEM can be built into GEM -PT NPs simultaneously with other hydrophobic antitumor medications, such as GEM and PT. GEM was preferred for this study and its centers were embodied into GEM-PT NPs close to PT, because of its cooperative energy with PT. The main procedure of stacking of GEM and PT inside GEM-PT NPs is shown in Figure 1. GEM and PT are incorporated in the polymer framework of GEM-PT NPs done by hydrophobic interaction. Hence, the insertions are restricted by similarities concerning GEM and PT and their hydrophobic interaction with the co-polymer. Self-assembled nanoparticles (GEM-PT NPs) were formed spontaneously with 4 mg/ml PT and 8 mg/ml GEM by employing intermolecular hydrophobic interactions between the lipophilic core of GEM and PT, as depicted in Figure 1.

**Figure 2.**
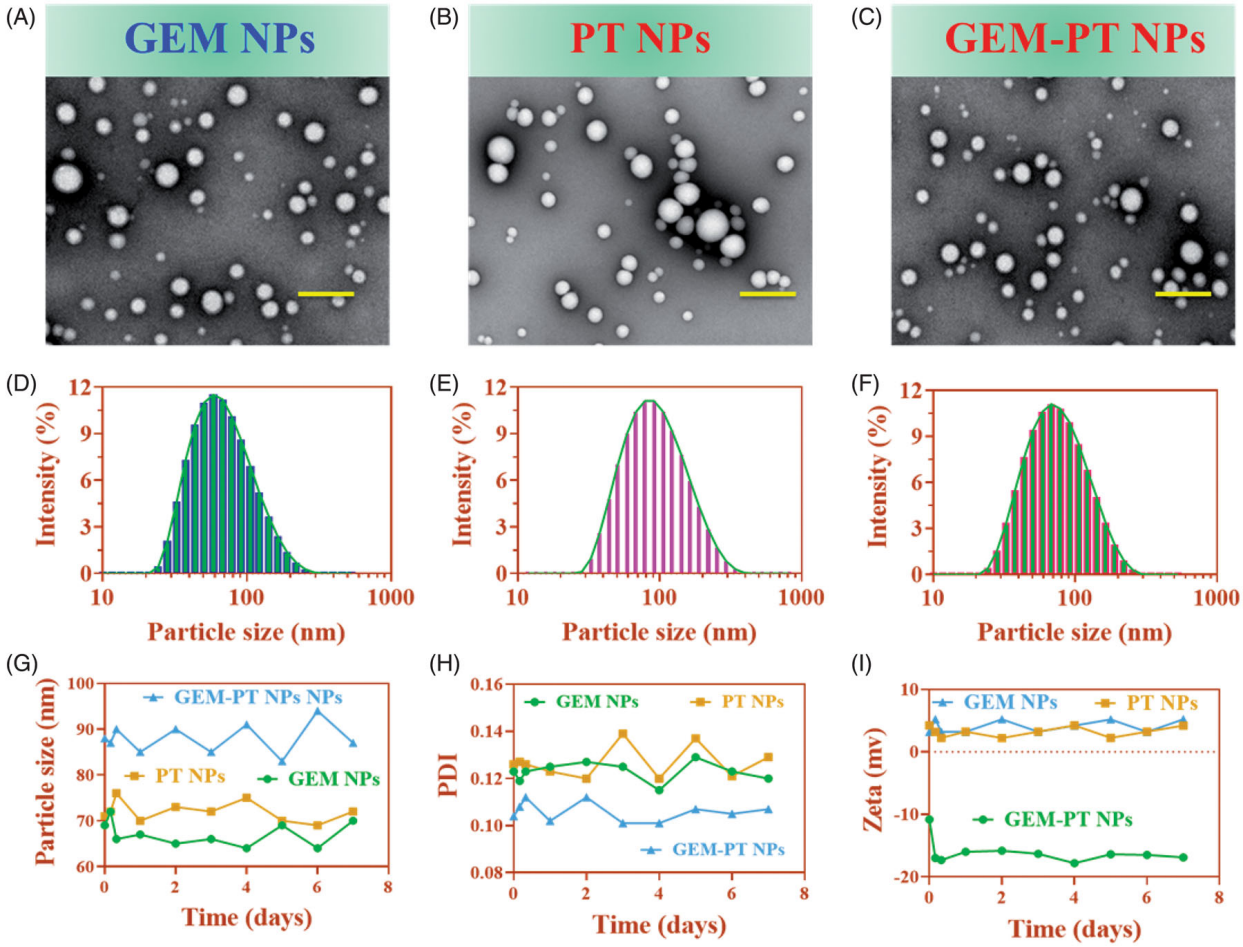
Characterization of the nanoparticles. (A–F) Morphology and particle size of GEM NPs, PT NPs, and GEM-PT NPs under a transmission electron microscope after negative staining with sodium phosphotungstate solution (2%, w/v). Scale bar: 20 nm. Particle size distribution of GEM NPs, PT NPs, and GEM-PT NPs analyzed by dynamic light scattering via a Zetasizer. (G–I) Stability of the GEM NPs, PT NPs, and GEM-PT examined by the dynamic light scattering.

The effects of the morphological surface of the hydrothermally prepared GEM NPs, PT NPs, and GEM-PT NPs were investigated through TEM analysis (Zhang et al., 2013; Wu et al., 2020; Yalcin et al., 2020). The results as shown in Figure 2(A–C) depicts the creation of GEM-PT NPs. Additionally, morphological changes the synthesized polymeric NPs were analyzed by HR-TEM. The nanocomposite was composed of from agglomerated clusters of well-shaped hydroxyapatite nanocomposites (Figure 2(A–C)). The size of the GEM-PT NPs was examined by dynamic light scattering (DLS) analysis. The diameters of GEM NPs, PT NPs, and GEM-PT NPs measured from TEM images were in the range of 63.8 ± 2.3, 69.3 ± 1.8, and 83.2 ± 1.9 nm (Figure 2(D–F)) and the Polyplexes index were 0.277 ± 0.05, 0.252 ± 0.05, and 0.159 ± 0.02 for GEM NPs, PT NPs, and GEM-PT NPs, respectively, which is in agreement with the results of light scattering measurements and gives clear evidence of the size of the NPs compared to those analyses by TEM (Figure 2(D–F)). The stability of the GEM NPs, PT NPs, and GEM-PT NPs in PBS media was examined by determining the particle size of the GEM NPs, PT NPs, and GEM-PT NPs by DLS. Polyplexes index, specifically GEM NPs, PT NPs, and GEM-PT NPs, at an NPs ratio of 100:1 were organized and incubated for 30 min at 37 °C in order to confirm complete polyplex formation (Figure 2(G–H)). All the experiments were repeated three times. Additionally, the zeta potential and the stability of GEM NPs, PT NPs, and GEM-PT was determined to be 5.2 ± 0.4, 6.8 ± 0.5, and −6.3 ± 0.3 mV (Figure 2(I)) by DLS. Hence, this fabrication approach for GEM-PT NPs produced favorable particle sizes, which may potentially increase intratumoral accumulation. In addition, the values of EE and the percentages of DL was determined by HPLC analysis. As a result of 1:1 ratio of the GEM and PT, the EE values were 91.0 ± 0.8 and 95.0 ± 2.0% for GEM and PT, respectively. The percentage of DL were 4.3 and 9.5% for GEM-PT NPs, respectively.

### 3.2. Controlled release of GEM-PT NPs

**Figure 3.**
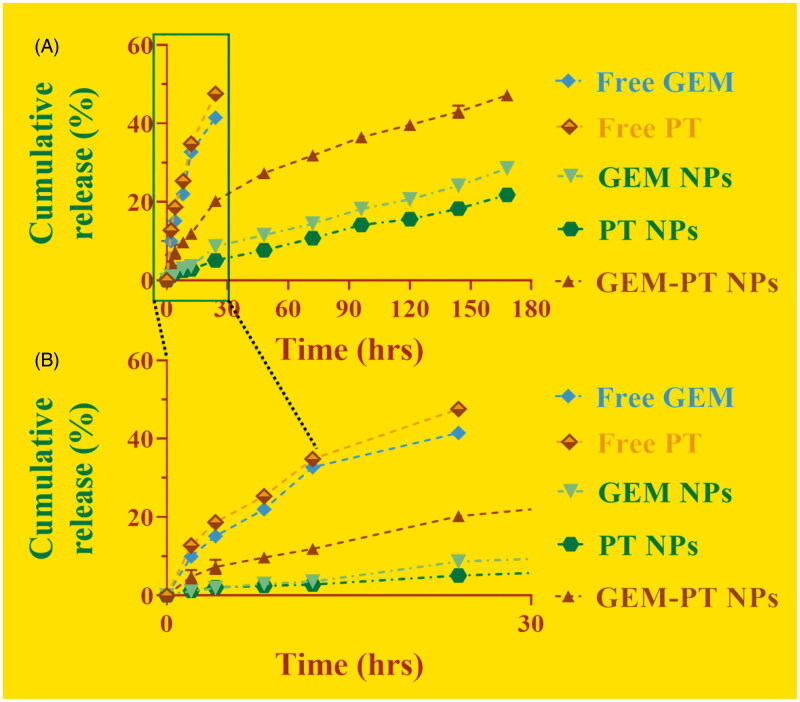
(A) Drug release profiles (GEM and PT) from the GEM NPs, PT NPs, and GEM-PT NPs against PBS containing 0.3% polysorbate 80%. (B) Enlarged figure of drug release profiles (GEM and PT) from the GEM NPs, PT NPs, and GEM-PT NPs.

Controlled release of GEM-PT NPs plays a vital role in the size, solubility, degradation, and drug loading by the NP frameworks. It is predictable results to confirm the drug release profile shows the PT and GEM-loaded GEM-PT NPs reserve an enhanced efficiency to the frameworks. In contrast, if the drugs not deceived, a reckless and undesired untimely discharge will occur. These methods provide clues to the production of shell holes that permit the discharge of drugs (Delplace et al., 2014; Tabatabaei Rezaei et al., 2015; Llinàs et al., 2018). The controlled drug release was measured via physical and chemical analysis of the GEM-PT NPs and the encapsulation properties of the drugs. These dialysis methods were utilized to examine the outcomes of controlled release of the drugs encapsulated in the GEM-PT NPs and those associated with the Free PT and GEM. The controlled release experiment was conducted in PBS at a pH of 7.4 at 37 °C. The controlled release profiles of the combination of PT and GEM loaded in the GEM-PT NPs displayed an initial release in about 5 h monitored via sluggish release for six days (Figure 3(A,B)). First 10 h, half of the PT and GEM was discharged after the GEM-PT NPs formations. Subsequently, later 24 h, a gentle release of 40–50% was observed. These results indicate that the conjugation of PT and GEM on the surface of the PLGA-NPs (GEM-PT NPs) did not show any adverse effect on the controlled release by these nanocomposites.

### 3.3. *In vitro* cytotoxicity

**Figure 4.**
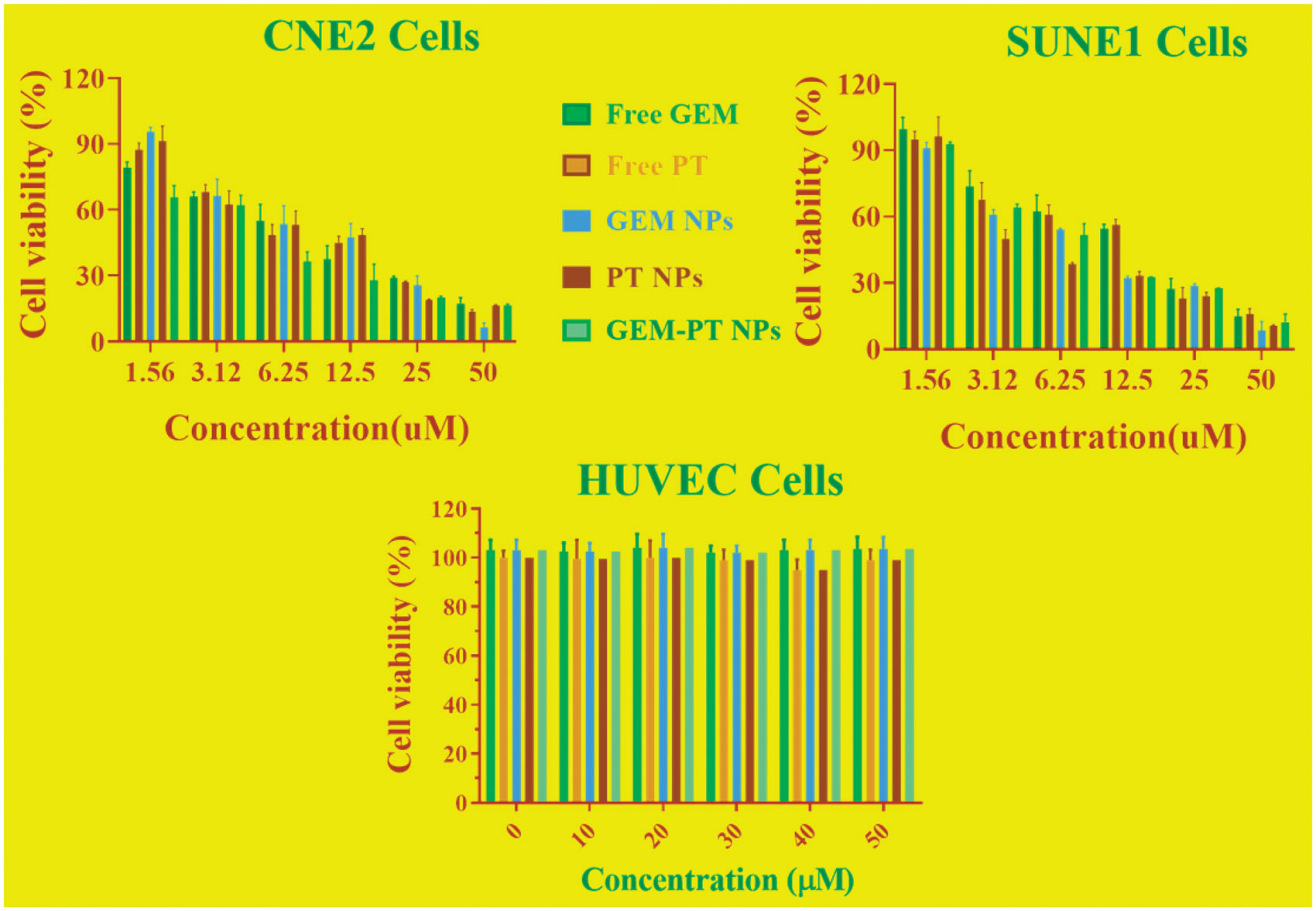
*In vitro* cytotoxicity of free PT, free GEM, PT NPs, GEM NPs, and GEM-PT NPs were evaluated in CNE2 and SUNE1 cancer cells. Cell viability was examined by the MTT assay after 24 h of drug incubation. Cell viability of non-cancerous HUVEC cells after treatments with different samples for 24 h.

After successful fabrications of GEM-PT NPs, we performed an MTT assay to evaluate the cytotoxic effects of Free PT, Free GEM, PT NPs, GEM NPs, and GEM -PT NPs on cancer cell lines, comprising CNE2 and SUNE1 cancer cells. Following treatments with the drugs for 24 h, the cells viability was monitored, and minimum-inhibitory concentrations (IC_50_) were obtained from the dose-dependent curve (Figure 4). Surprisingly, GEM-PT NPs displayed substantial improvement in cytotoxicity of the cancer cells. For instance, in CNE2 cell lines, IC_50_ of 10.91 ± 11.12, 10.35 ± 1.22, 9.05 ± 2.11, 9.46 ± 0.98, and 6.62 ± 0.97 were observed for free PT, free GEM, PT NPs, GEM NPs, and GEM-PT NPs, respectively. In SUNE1 cell lines, IC_50_ of 19.27 ± 3.30, 17.70 ± 2.54, 11.20 ± 0.98, 10.22 ± 1.87, and 7.16 ± 2.80 for Free PT, Free GEM, PT NPs, GEM NPs, and GEM-PT NPs were observed, respectively. The enhanced cytotoxicity of the GEM-PT NPs was owing to the entire release of the double potential anticancer medications into the tumor cells. The hydrophilic molecules of PLGA dispense the aqueous layer via a lipid bilayer for cell membrane penetration. Thus, the enhancement of cellular uptake requires the cell membrane nucleosides delivery for the proteins. One of the major limitations of existing antitumor drugs is their poor selectivity for killing cancer cells over noncancerous cells, which usually causes side effects and impairs the dose intensification of drugs in clinic. To assess whether free PT, free GEM, PT NPs, GEM NPs, and GEM-PT NPs exert the activity as cancer-selective agents, we additionally tested the cytotoxicity in noncancerous cell line human umbilical vein endothelial HUVEC cells. Cisplatin exhibited the high cytotoxicity in both cells; the IC_50_ values in noncancerous cells are comparable with those in cancer cells (Figure 4). Interestingly, the free PT, free GEM, PT NPs, GEM NPs, and GEM-PT NPs were less toxic in HUVEC cells.

### 3.4. Morphological changes in CNE2 cancer cells

**Figure 5.**
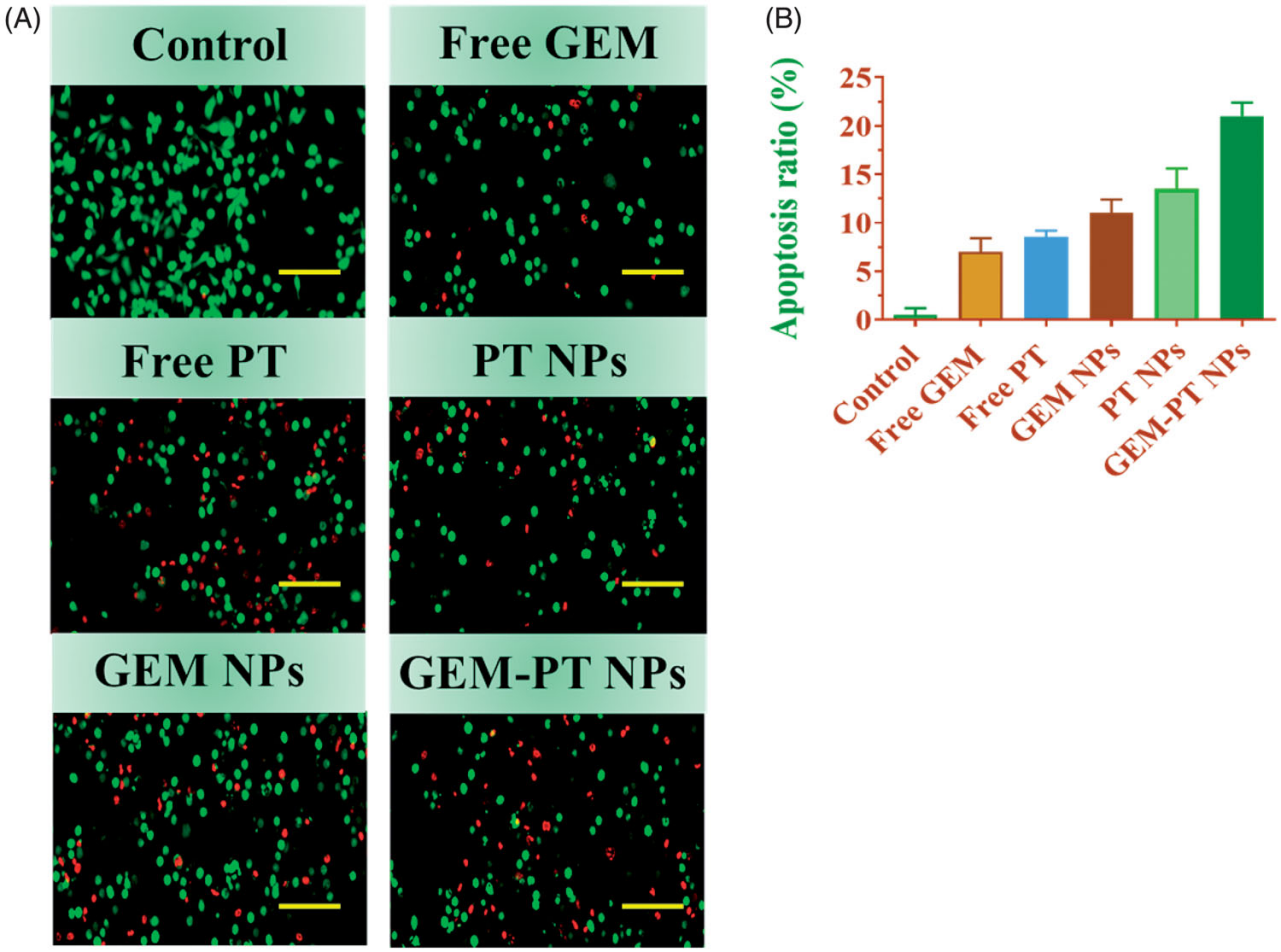
Dual AO/EB staining assay for examining Free PT, Free GEM, PT NPs, GEM NPs, and GEM-PT NPs-induced cell death in CNE2 cells. The cells were treated with Free PT, Free GEM, PT NPs, GEM NPs, and GEM-PT NPs at 2.5 µM concentration for 24 h. (B) Quantification of apoptosis ratio. The cells were quantified by image J software.

Dual staining AO-EB is a qualitative technique used to identify live, early, late apoptotic, and necrotic cancer cells using fluorescent images to observe morphological changes in the nucleus of cells. AO permeates the intacts membranes of usual and early apoptotic cell and binds to DNA, which fluoresces uniform green in normal cells and as patches in early apoptotic cells due to chromatin condensations (Li & Gao, 2020). In difference, EB is only penetrable in the incapacitated membrane of late apoptotics and necrotics cell, where it fluoresces as bright orange patch through its bindings to DNA fragment or apoptotic bodies in late apoptotic cells, and as a unchanging orange fluorescence in the necrotic cell, due to have the nuclear changes in the morphology of viable cell. AO-EB-stained CNE2 cells were incubated with Free PT, Free GEM, PT NPs, GEM NPs, and GEM-PT NPs for 24 h. As presented in Figure 5(A), the presence of orange with reddish fluorescence with chromatin fragmentation after treatment of CNE2 cells treated with Free PT, Free GEM, PT NPs, GEM NPs, and GEM-PT NPs suggested that the GEM-PT NPs largely induced apoptosis in CNE2 cells (Figure 5(B)).

**Figure 6.**
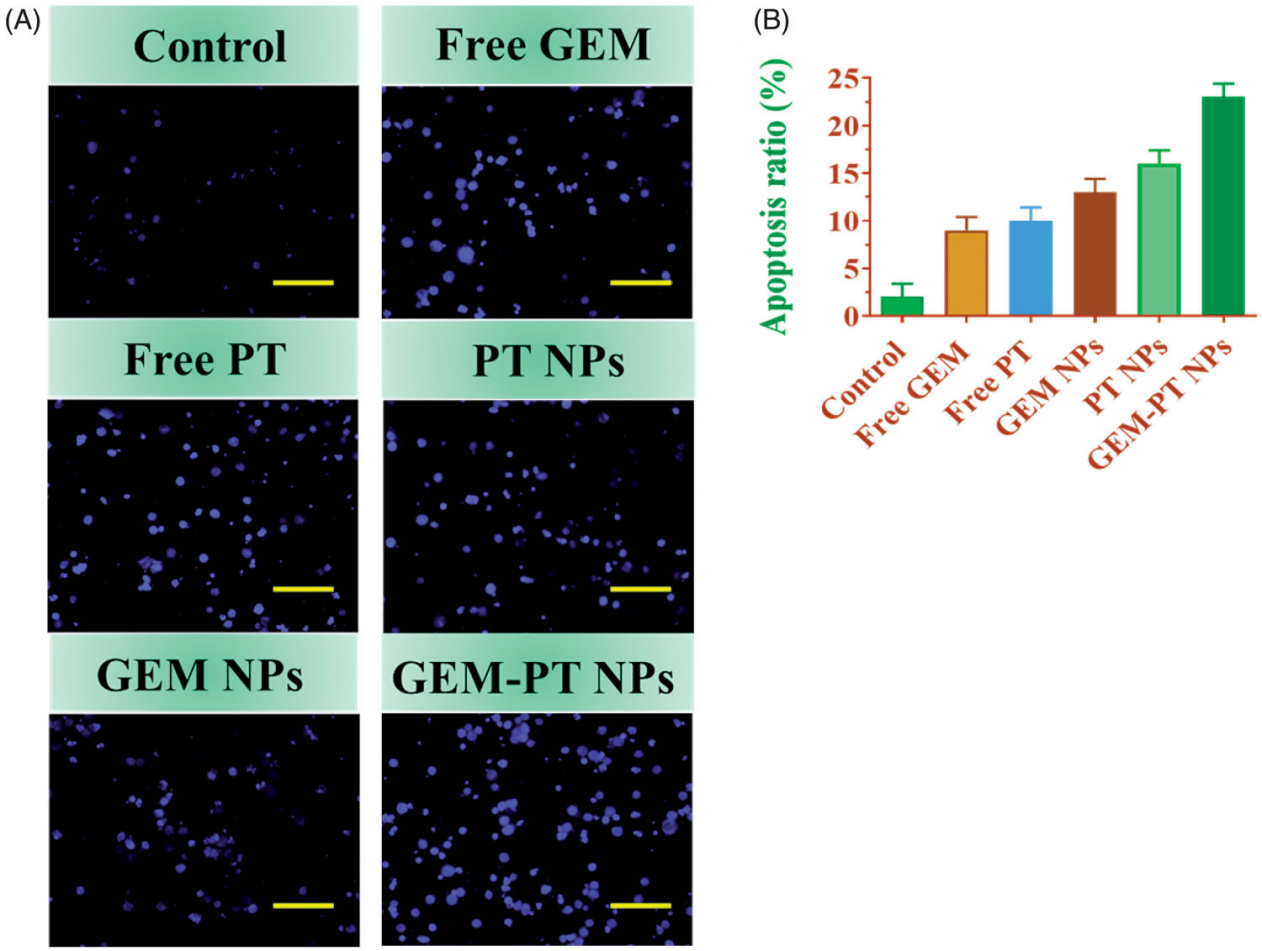
Nuclear (Hoechst 33258) staining assay for examining Free PT, free GEM, PT NPs, GEM NPs, and GEM-PT NPs-induced cell death in CNE2 cells. The cells were treated with Free PT, Free GEM, PT NPs, GEM NPs, and GEM-PT NPs at 2.5 µM concentration for 24 h. (B) Quantification of apoptosis ratio. The cells were quantified by image J software.

Hoechst 33258 staining was used to observe chromatin fragmentation, bi- and/or multinucleation, cytoplasmic vacuolation, nuclear swelling, cytoplasmic bleating, and late apoptosis in cancer cells by visualizing dot-like chromatin condensation. Hoechst-33258–stained CNE2 cells were incubated with Free PT, Free GEM, PT NPs, GEM NPs, and GEM-PT NPs for 24 h. As displayed in Figure 6(A), the presence of blue fluorescence with chromatin condensation after treatment of CNE2 cells treated with Free PT, Free GEM, PT NPs, and GEM NPs suggested that the GEM-PT NPs largely induced apoptosis in CNE2 Nasopharyngeal cancer cells (Figure 6(B)).

### 3.5. Apoptosis in CNE2 cancer cells

**Figure 7.**
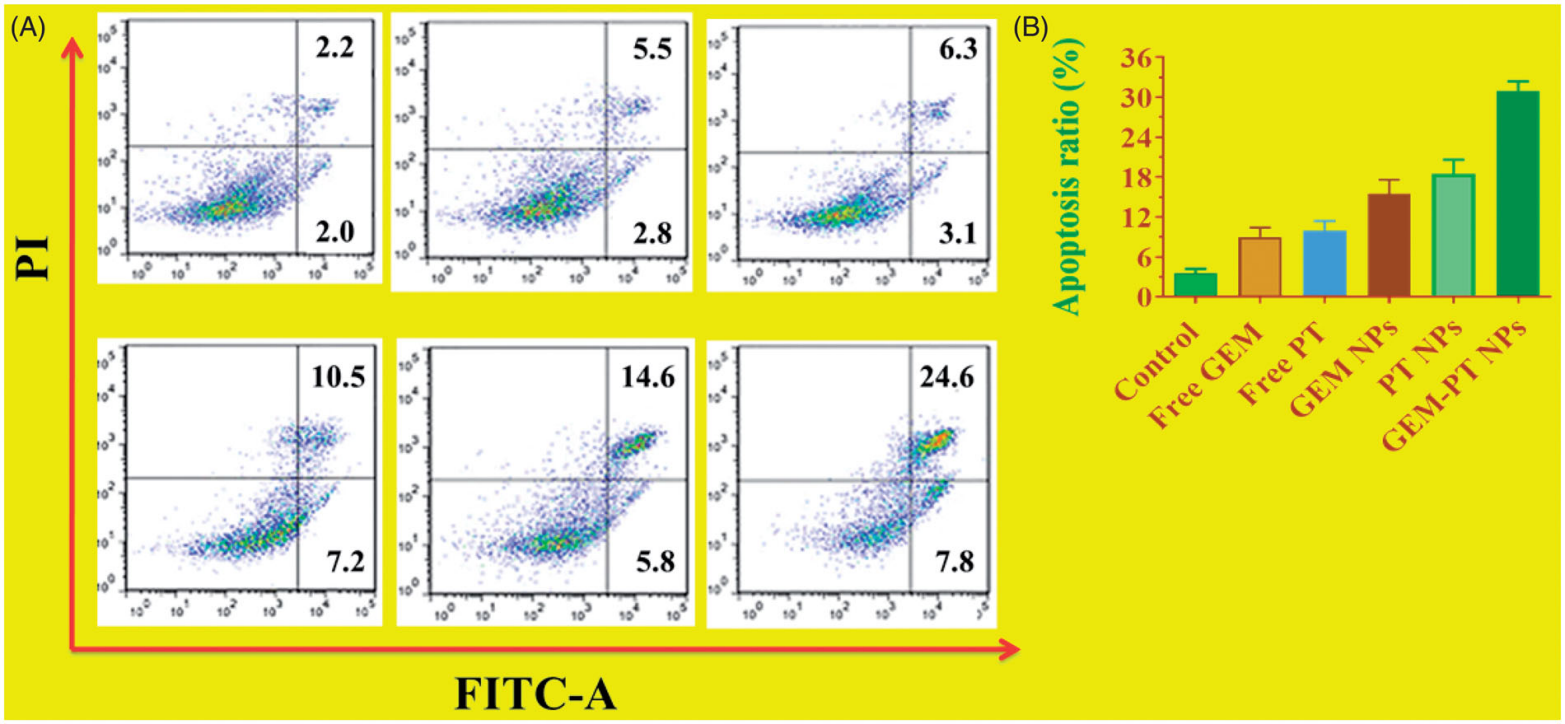
(A) Apoptotic analysis of CNE2 cells using flow cytometry. The cells were treated with free PT, free GEM, PT NPs, GEM NPs, and GEM-PT NPs at 2.5 µM concentration for 24 h and then stained with FITC annexin V/PI for flow cytometry analysis. (B) Apoptosis ratio of CNE2 cells.

Apoptosis may be reckoned as an important obstacle for a damaged cell to become malignant tumors. Since the complexes promote apoptosis induction in cancer cells, flow cytometry using Annexin V-FITC/propidium iodide (PI) double staining was carried out for the quantitative discrimination of apoptotic cells. Phosphatidylserine (PS) is a cell cycle signaling phospholipid located inner side of the membrane of a healthy cell but is reverted to the outer membrane for recognition by neighboring cells at the time of apoptosis (Tambe et al., 2018). Hence, the translocation of PS is a morphological hallmark of apoptosis and can be spotted by its binding with fluorescently labeled Annexin V which in turn detected by flow cytometry. Further the addition of PI to Annexin V stained cells is used to discriminate and concomitantly quantify the live cells (lower left quadrant-Annexin V(-)/PI(-)), early apoptotic cells (upper left quadrant-Annexin V(+)/PI(-)) and late apoptotic cells (upper right-quadrant-Annexin V(+)/PI(+)) using FACS. As projected in Figure 7(A), the incubation of Free PT, Free GEM, PT NPs, GEM NPs, and GEM -PT NPs with CNE2 cells conspicuously induced apoptosis. It is worth to note that the titled complexes induce apoptosis even at very low concentrations which is less than their IC_50_. In comparison with control, the cell population was higher (6–9%) in Annexin V(+)/PI(-) (upper left) quadrant indicating the induction of early apoptosis (Figure 7(B)). This effect was ascertained to be high for GEM -PT NPs than the Free PT, Free GEM, PT NPs, GEM NPs analogous with the results of MTT, and AO-EB staining assays. It is to note that the test samples displayed comparatively better apoptotic induction on CNE2 cells.

### 3.6. Histological evaluation for systemic toxicity

**Figure 8.**
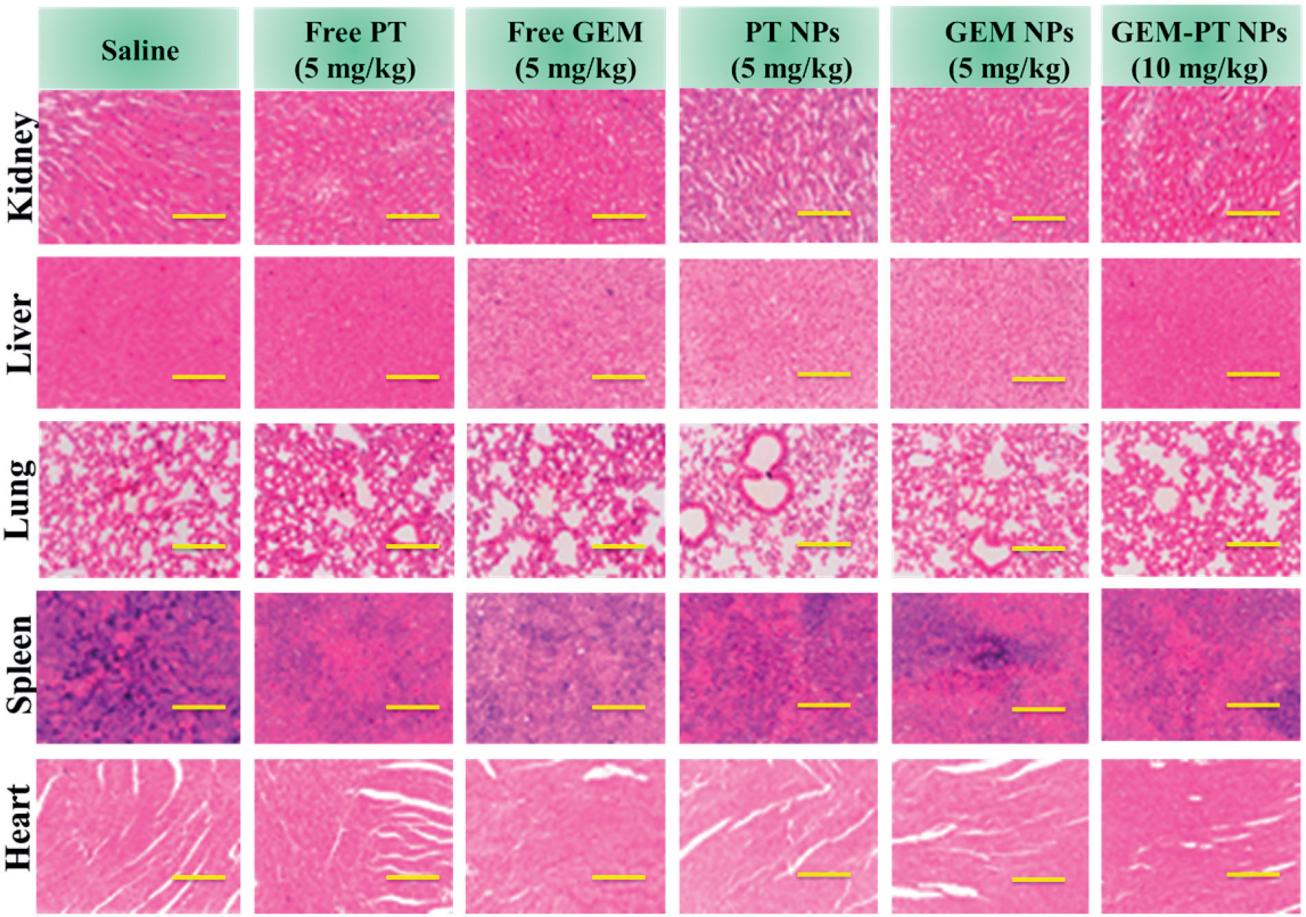
H&E staining of the major organs (kidney, liver, lung, spleen, and heart) excised from different treatment mice groups. Scale bar: 50 μm.

The efficiency of anticancer chemotherapeutic drugs is mainly validated by its selective action toward cancer tissues leaving the normal organs undamaged. After the verification of low systemic toxicity in the mice injected with Free PT (2.5 and 5 mg/kg), Free GEM (2.5 and 5 mg/kg), PT NPs (2.5 and 5 mg/kg), GEM NPs (2.5 and 5 mg/kg), and PT-GEM NPs (2.5, 5, and 10 mg/kg), histological analyses were carried out to identify the structural changes in the tissues of vital of organs inclusive of heart, liver, spleen, lung, and kidney of the mice treated with Free PT, Free GEM, PT NPs, GEM NPs, and PT-GEM NPs and compared with control, the saline received mice. Figure 8 represented the histological sections of the heart, liver, spleen, lung, and kidney stained with hematoxylin and eosin (H&E). The photomicrographs of the liver and spleen of the control, Free PT, Free GEM, PT NPs, GEM NPs, and PT-GEM NPs treated groups displayed normal cellular morphology. Under optical microscopy examination, the heart, lung, and kidney of Free PT, Free GEM, PT NPs, GEM NPs, and PT-GEM NPs treated animals showed normal cardiac muscle fibers, normal alveolar, and normal glomerular histological characteristics respectively which were found to be similar histological architecture as those of the control group with no treatment-related inflammatory response.

### 3.7. *In vivo* antitumor efficacy in CNE2 xenograft tumor model

**Figure 9.**
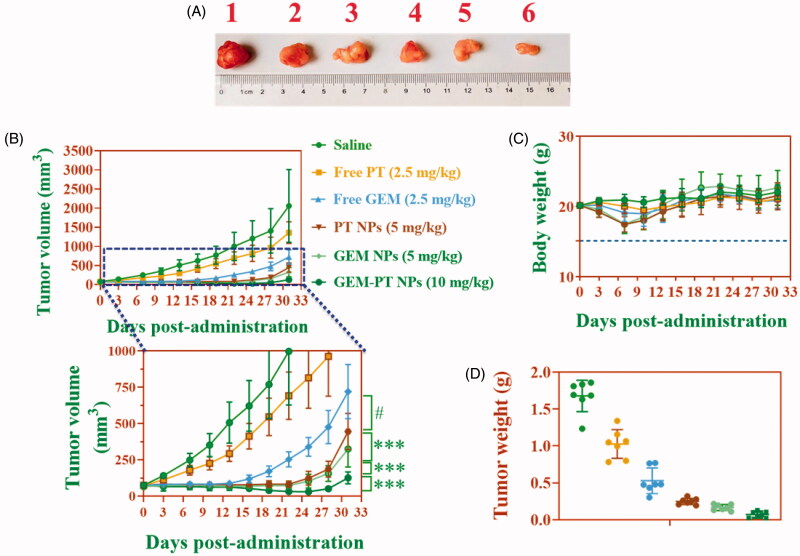
*In vivo* antitumor activity of free PT, free GEM, PT NPs, GEM NPs, and PT-GEM NPs compared to saline. CNE2 tumor xenograft-bearing Balb/c nude mice were administered with various drugs via intravenous injection at days 0, 3, and 6. (A) Changes in tumor volumes. (B) Body weights. (C) Represent tumor photograph. (D) Tumor weights. The data are presented as the means ± SD (*n* = 7).

Considering the promising *in vitro* biological activity profiles, the *in vivo* pharmacological efficacy was further investigated in a CNE2 xenograft tumor model. In the experimental process, body weight of animals in each group was stable. It suggested that the experimental doses in all groups were tolerable. As shown in Figure 9(A–C), we found an obvious retardation of tumor growth for animals treated with Free PT, Free GEM, PT NPs, GEM NPs, and PT-GEM NPs, as compared to the control group. Specifically, NPs delivering PT-GEM NPs more efficiently suppressed tumor growth than administered free PT, free GEM (Figure 9) panels a tumor site(s) via the EPR effect. Moreover, these PT-GEM NPs did not significantly affect the body weights of mice, indicating that the delivery materials and Free GEM have low systemic toxicity. Most importantly, treatment with the combination of PT-GEM NPs could significantly enhance the efficacy of chemotherapy for PT-GEM NPs, as evidenced by more remarkable slow-down for tumor growth in relative to the Free PT, Free GEM, PT NPs, and GEM NPs group (*P* < 0.05). On day 33, animals in saline groups performed a high average tumor weight of 1.58 g (Figure 9(D)). The animals treated with Free PT, Free GEM, PT NPs, GEM NPs, and PT-GEM NPs exhibited lower mean tumor weight of 0.92, 0.50, 0.36, 0.29, and 0.09 g, respectively. A significantly lower mean tumor weight was obvious for PT-GEM NPs compared to free PT, free GEM (*p* < .05).

## 4. Conclusion

We developed GEM-PT NPs by encapsulating GEM and PT core to change the tumor microenvironment for improved drug accretion and additional anticancer activities. At first, PT was incorporated into GEM-PT NPs with effectual loading and encapsulation by direct self-assembly method. In this study, we showed that PT could be made hydrophobic by using an oil/water solvent evaporation method for drug delivery. These DOPA-covered PT centers were compatible with macromolecular PLGA and could be coencapsulated in GEM-PT NPs. The closeness of the PT centers fundamentally developed the epitome of GEM into PLGA-NPs. The formation of the nanocomposite was confirmed by TEM electroscopic techniques displayed the crystallized structure of the nanocomposite. GEM-PT NPs comprising double PT and GEM led to remarkable apoptosis in human Nasopharyngeal CNE2 and SUNE1 cancer cells. Further, morphological changes in the cells were monitored using dual staining and nuclear staining methods. AO-EB fluorescent staining and flow cytometry analysis reveal that the samples induce cancer cell death by apoptosis mechanism. Moreover, *in vivo* investigation in a CNE2 xenograft tumor model demonstrated the outstanding antitumor efficacy of GEM-PT NPs significantly in superior to the rest of the samples. In summary, the results of this study demonstrated that GEM-PT macromolecular NPs for local delivery as a novel combination strategy may enhance the therapeutic potency for the treatment and nursing care of nasopharyngeal cancer, and has promising clinical implications in future.
